# Identification of discriminative characteristics for clusters from biologic data with InforBIO software

**DOI:** 10.1186/1471-2105-8-281

**Published:** 2007-08-02

**Authors:** Naoto Tanaka, Masataka Uchino, Satoru Miyazaki, Hideaki Sugawara

**Affiliations:** 1 Center for Information Biology and DDBJ, National Institute of Genetics, 1111 Yata, Mishima, Shizuoka 411-8540, Japan; 2 Institute for Bioinformatics Research and Development (BIRD), Japan Science and Technology Corporation (JST), 5-3 Yonbancho, Chiyoda-ku, Tokyo 102-8666, Japan; 3 Laboratory of Information Biology, Faculty of Pharmaceutical Science, Tokyo University of Science, 2641 Yamazaki, Noda Chiba 278-8510, Japan; 4 Department of Applied Biology and Chemistry, Tokyo University of Agriculture, 1-1-1 Sakuragaoka, Setagaya-ku, Tokyo 156-8502, Japan; 5 SOKENDAI, Hayama, Kanagawa 240-0193, Japan

## Abstract

**Background:**

There are a number of different methods for generation of trees and algorithms for phylogenetic analysis in the study of bacterial taxonomy. Genotypic information, such as SSU rRNA gene sequences, now plays a more prominent role in microbial systematics than does phenotypic information. However, the integration of genotypic and phenotypic information for polyphasic studies is necessary for the classification and identification of microbes. Thus, we devised an algorithm that objectively identifies discriminative characteristics for focused clusters on generated trees from a dataset composed of coded data, such as phenotypic information. Moreover, this algorithm has been integrated into the polyphasic analysis software, InforBIO.

**Results:**

We developed a differential-character-finding algorithm based on information measures and used this algorithm to identify the characteristic that best discriminates operational taxonomic unit clusters. For all characteristics in a dataset, the algorithm estimates commonality in focused clusters and diversity among clusters by scoring based on Shannon's and relative entropies. All the characteristics selected for scoring are equally weighted. Thresholds for the scores are defined to identify discriminative characteristics for clusters efficiently from a database. The unique feature of the algorithm, which is implemented in the InforBIO software, is that it can identify the phenotypic characteristics that discriminate and are associated with the clusters of a phylogenetic tree. We successfully applied this algorithm to the study of phylogenetic clusters of *Pseudomonas *species.

**Conclusion:**

The algorithm in the InforBIO software is a novel and useful approach for microbial polyphasic studies. The algorithm can also be applied to diverse cluster analyses. The InforBIO software is available from the download site . This software is free for personal but not commercial use.

## Background

It is common practice for biologists to apply cluster analysis to both genotypic and phenotypic data of operational taxonomic units (OTUs). Genotypic data, primarily small subunit ribosomal RNA (rRNA) gene sequences and DNA-DNA similarities based on the hybridization technique, play a more prominent role than do phenotypic data in current microbial systematics [1,2]. Phylogenetic analysis based on single gene sequences is useful for understanding relations between species but is rarely used to define species. DNA-DNA similarities based on the hybridization technique are recommended for species definition, and the cut-off is defined [2]. However, it is actually and technically difficult to conclude when a similarity is close to the defined cut-off. In contrast, phenotypic data are still important for efficient identification and recognition of biologic features and can be obtained with kits such as API (bioMerieux, Inc., Lyon, France) and BiOLOG (Biolog, Inc., Hayward, CA, USA). In 2002, the ad hoc committee of the International Committee for the Systematics of Prokaryotes [3] made the following recommendations with respect to the value of phenotypes in a species description: "(1) Species should be identifiable by readily available methods (phenotypic and genotypic). Efforts should be made to establish standardized methods of reporting phenotypic and genomic data; (2) Minimal characteristics should be provided...; and (3) Phenotype, including chemotaxonomic markers, will remain important diagnostic properties in a species description." These recommendations can be fully addressed only through polyphasic studies based on the integration of genotypic and phenotypic data [4,5]. An information system for polyphasic studies is needed for the classification and identification of microbes.

A number of algorithms and programs for clustering and generating trees have been developed for numerical and phylogenetic analyses in bacterial taxonomy [6,7]. It is often difficult to identify phenotypic characteristics that can discriminate clusters defined on the bases of gene sequences. At present, if it is difficult to identify discriminative characteristics for a species from available phenotypic data, the species is studied on the basis of the genomovars concept as was reported for *Pseudomonas stutzeri *strains [8], although cryptic discriminative characteristics may be found. It can be difficult to identify discriminative characteristics in the case of a large and diverse phenotypic dataset.

We developed the InforBIO software for the study of microbial diversity [9], and the software is freely available from the download site [10]. The user can seamlessly repeat a workflow from data management, data analysis, and evaluation of analytical results. The software includes functions for data handling, including design of databases, storage and retrieval of data, numerical analysis, phylogenetic analysis, and discriminative and probabilistic identification. All the features of the InforBIO software are applicable to any biologic object from molecules to organisms, if the data are coded in the same way as microbial data.

In the present study, we devised a differential-character-finding algorithm that objectively identifies discriminative characteristics for focused clusters from a dataset composed of coded data, and this algorithm was integrated into the InforBIO software.

## Implementation

The differential-character-finding algorithm consists of two types of mathematical measures based on Shannon's entropy [11] and relative entropy [12]. We named these measures "common score" and "differential score", respectively. With our differential-character-finding algorithm, the following events are executed in the InforBIO software: 1) construction of a database of biologic data, including coded and sequence data; 2) construction of phylogenetic trees (or numeric dendrograms) from datasets in the database; 3) selection of target clusters for differential-character-finding analysis on the phylogenetic tree (or numeric dendrograms); 4) calculation of common and differential scores for each characteristic on target clusters; and 5) identification of the most discriminative characteristic(s) for each target cluster with reference to thresholds for the two scores depending on the range of scores for the value.

### InforBIO

A biologic database and analysis programs are integrated into the InforBIO software, and the system architecture was described previously [9]. An ID number is automatically assigned to an OTU in a biologic database. Each phenotypic characteristic (e.g., assimilation of glucose) of an OTU is described in a biologic database by a testable variable associated with the characteristic. In general, most tests take the character data type as the test's result, such as "+++", "+", and "-". Therefore, we consider the complete event system for each item variable to calculate the common and differential scores. We can then calculate the occurrence probabilities of every value for the item variable on the complete event systems. In the InforBIO software, up to 12 values (e.g., +) can be assigned for a test item (e.g., glucose assimilation). The InforBIO software can also manage multiple gene and protein sequence data of OTUs on a database.

**Figure 1:**
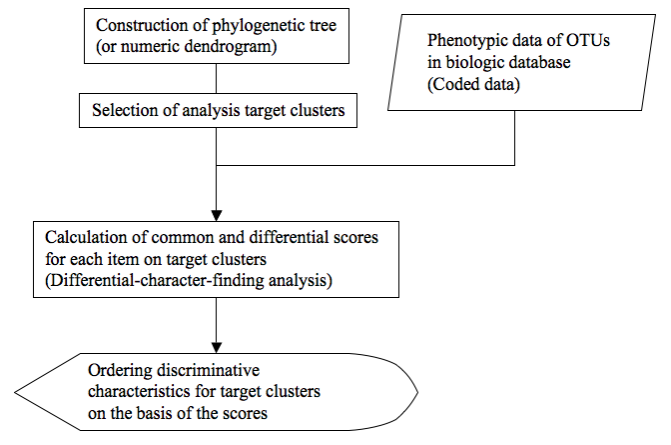
Flowchart of the differential-character-finding algorithm in the InforBIO software.

Phylogenetic trees, based on sequence data of OTUs from a biologic database, are constructed by programs in the InforBIO software. The name of an OUT should contain the ID number and species name. DNA and protein sequence data of OTUs are aligned with the ClustalW program [13] in the InforBIO software, and phylogenetic trees are then generated by either the ClustalW program [13] by the neighbor-joining method or the PHYLIP package [14,15] with either the maximum-likelihood (the DNAML program for DNA sequences and the PROML program for protein sequences) or the maximum-parsimony (the DNAPARS program for DNA sequences) methods. Additionally, the InforBIO software can import and process alignment and tree files from outside. Importable tree file format is Phylip, whereas alignment file formats are Clustal, Fasta, Phylip, GCG, GDE, and PIR, provided from other analysis programs such as MAFFT [16] and MUSCLE [17]. After generating a phylogenetic tree, users select target clusters on a generated tree for the differential-character-finding analysis and retrieve discriminative characteristics for the target clusters from the biologic database computationally with the differential-character-finding algorithm. Then, phenotypic data of OTUs in target clusters are retrieved with reference to each OUT name composed of ID number and species name. Thus, discriminative characteristics for clusters on trees generated by using outside data can be also analyzed in the InforBIO software when the OUT names are defined by the same format as described above. In addition, discriminative characteristics for clusters on a numeric dendrogram that is based on coded data of OTUs can be identified by the same manner as those for clusters on a phylogenetic tree in the InforBIO software. Numeric dendrograms are generated with either similarities or distances by the unweighted pair-group method with arithmetic mean and the neighbor-joining method. Similarities between OTUs are calculated from coded data with the simple matching, the Jaccard, and the dice coefficients, whereas distances are based on the euclidean distance. The flowchart to identify discriminative characteristics for target clusters in the InforBIO software is shown in figure 1.

### Differential-Character-Finding Algorithm

#### Common score

The common score is based on Shannon's entropy [11] and represents the randomness of the probabilistic distribution of *n *values (*n *≥ 2) between OTUs in a cluster. Thus, the common score of a discriminative item should be close or equal to 0 and is calculated as

Ccluster=∑n{−p(m)ln⁡[p(m)]}
 MathType@MTEF@5@5@+=feaafiart1ev1aaatCvAUfKttLearuWrP9MDH5MBPbIqV92AaeXatLxBI9gBaebbnrfifHhDYfgasaacH8akY=wiFfYdH8Gipec8Eeeu0xXdbba9frFj0=OqFfea0dXdd9vqai=hGuQ8kuc9pgc9s8qqaq=dirpe0xb9q8qiLsFr0=vr0=vr0dc8meaabaqaciaacaGaaeqabaqabeGadaaakeaacqWGdbWqdaWgaaWcbaGaem4yamMaemiBaWMaemyDauNaem4CamNaemiDaqNaemyzauMaemOCaihabeaakiabg2da9maaqahabaWaaiWaaeaacqGHsislcqWGWbaCcqGGOaakcqWGTbqBcqGGPaqkcyGGSbaBcqGGUbGBdaWadaqaaiabdchaWjabcIcaOiabd2gaTjabcMcaPaGaay5waiaaw2faaaGaay5Eaiaaw2haaaWcbaaabaGaemOBa4ganiabggHiLdaaaa@4D33@

where *p*(*m*) denotes the frequency (0 ≤ *p*(*m*) ≤ 1) of the occurrence probability of a value '*m*' for an item. The average common score of an item among defined clusters is represented as *C *in this study.

The upper threshold for *C*, *C*_*thr*_, is calculated by

Cthr=−aln⁡a−(n−1)(1−an−1)ln⁡(1−an−1)
 MathType@MTEF@5@5@+=feaafiart1ev1aaatCvAUfKttLearuWrP9MDH5MBPbIqV92AaeXatLxBI9gBaebbnrfifHhDYfgasaacH8akY=wiFfYdH8Gipec8Eeeu0xXdbba9frFj0=OqFfea0dXdd9vqai=hGuQ8kuc9pgc9s8qqaq=dirpe0xb9q8qiLsFr0=vr0=vr0dc8meaabaqaciaacaGaaeqabaqabeGadaaakeaacqWGdbWqdaWgaaWcbaGaemiDaqNaemiAaGMaemOCaihabeaakiabg2da9iabgkHiTiabdggaHjGbcYgaSjabc6gaUjabdggaHjabgkHiTiabcIcaOiabd6gaUjabgkHiTiabigdaXiabcMcaPiabcIcaOmaalaaabaGaeGymaeJaeyOeI0IaemyyaegabaGaemOBa4MaeyOeI0IaeGymaedaaiabcMcaPiGbcYgaSjabc6gaUjabcIcaOmaalaaabaGaeGymaeJaeyOeI0IaemyyaegabaGaemOBa4MaeyOeI0IaeGymaedaaiabcMcaPaaa@5276@

where *a *is an acceptable frequency of the occurrence probability (0.5 <*a *< 1) of a value for an item within a cluster. (1-*a*)/(*n*-1) indicates that the total of frequencies of the occurrence probability of other values for the item are divided by the number of other values. Hence, *C*_*thr *_is the maximal common score in the case of a defined *a*. The *C *for discriminative items should satisfy the condition of *C *≤ *C*_*thr*_.

#### Differential score

The differential score, *D*_*cluster*_, of an item, which is based on the relative entropy [12], represents the degree of difference between two probabilistic distributions within clusters (A and B). A discriminative item should, accordingly, have a high differential score. The score between two clusters, *D*_*cluster*_, is calculated as

Dcluster=12[Dcluster(A|B)+Dcluster(B|A)]=12[∑np(m)ln⁡p(m)q(m)+∑nq(m)ln⁡q(m)p(m)]
 MathType@MTEF@5@5@+=feaafiart1ev1aaatCvAUfKttLearuWrP9MDH5MBPbIqV92AaeXatLxBI9gBaebbnrfifHhDYfgasaacH8akY=wiFfYdH8Gipec8Eeeu0xXdbba9frFj0=OqFfea0dXdd9vqai=hGuQ8kuc9pgc9s8qqaq=dirpe0xb9q8qiLsFr0=vr0=vr0dc8meaabaqaciaacaGaaeqabaqabeGadaaakeaacqWGebardaWgaaWcbaGaem4yamMaemiBaWMaemyDauNaem4CamNaemiDaqNaemyzauMaemOCaihabeaakiabg2da9maalaaabaGaeGymaedabaGaeGOmaidaamaadmaabaGaemiraq0aaSbaaSqaaiabdogaJjabdYgaSjabdwha1jabdohaZjabdsha0jabdwgaLjabdkhaYbqabaGccqGGOaakcqWGbbqqcqGG8baFcqWGcbGqcqGGPaqkcqGHRaWkcqWGebardaWgaaWcbaGaem4yamMaemiBaWMaemyDauNaem4CamNaemiDaqNaemyzauMaemOCaihabeaakiabcIcaOiabdkeacjabcYha8jabdgeabjabcMcaPaGaay5waiaaw2faaiabg2da9maalaaabaGaeGymaedabaGaeGOmaidaamaadmaabaWaaabCaeaacqWGWbaCcqGGOaakcqWGTbqBcqGGPaqkcyGGSbaBcqGGUbGBdaWcaaqaaiabdchaWjabcIcaOiabd2gaTjabcMcaPaqaaiabdghaXjabcIcaOiabd2gaTjabcMcaPaaaaSqaaaqaaiabd6gaUbqdcqGHris5aOGaey4kaSYaaabCaeaacqWGXbqCcqGGOaakcqWGTbqBcqGGPaqkcyGGSbaBcqGGUbGBdaWcaaqaaiabdghaXjabcIcaOiabd2gaTjabcMcaPaqaaiabdchaWjabcIcaOiabd2gaTjabcMcaPaaaaSqaaaqaaiabd6gaUbqdcqGHris5aaGccaGLBbGaayzxaaaaaa@8BFE@

where *p*(*m*) and *q*(*m*) denote the frequencies of the occurrence probability of a value '*m*' for an item in OTUs in clusters A and B, respectively. Each frequency should be more than 0 and less than 1. When more than two clusters are defined, each cluster is compared individually with every other cluster. The average of the resulting *D*_*cluster *_scores is defined as the differential score, *D*, of the item, which is calculated by

D=TS[y(y−1)2]
 MathType@MTEF@5@5@+=feaafiart1ev1aaatCvAUfKttLearuWrP9MDH5MBPbIqV92AaeXatLxBI9gBaebbnrfifHhDYfgasaacH8akY=wiFfYdH8Gipec8Eeeu0xXdbba9frFj0=OqFfea0dXdd9vqai=hGuQ8kuc9pgc9s8qqaq=dirpe0xb9q8qiLsFr0=vr0=vr0dc8meaabaqaciaacaGaaeqabaqabeGadaaakeaacqWGebarcqGH9aqpdaWcaaqaaiabdsfaujabdofatbqaamaadmaabaWaaSaaaeaacqWG5bqEcqGGOaakcqWG5bqEcqGHsislcqaIXaqmcqGGPaqkaeaacqaIYaGmaaaacaGLBbGaayzxaaaaaaaa@3AAC@

where *y *indicates the number of defined clusters. The denominator indicates the number of combinations of all clusters, and the numerator indicates a total score (*TS*) of the differential scores.

The significant lower threshold for *D*, *D*_*thr*_, is determined by the substitution of *TS *in formula (4) with (*y*-1)*D*_*cluster*_, where *D*_*cluster *_is calculated with an acceptable frequency of the occurrence probability *a *(0.5 <*a *< 1) on the basis of formula (3). The four frequencies are then substituted as *p*(*m*) = *a*, *p*(*n*) = 1-*a*, *q*(*m*) = 0.000001, and *q*(*n*) = 0.999999, respectively. Hence, *D*_*thr *_is calculated in the same manner as the *D *score of an item assigned two values (*n *= 2). The *D *for discriminative items should satisfy the condition of *D*_*thr *_≤ *D*.

Consequently, the common score is a useful measure of the commonality of characteristics of OTUs in a cluster, whereas the differential score is a measure of the differences between clusters. Their thresholds are effective for the rejection of unsuitable items for the discrimination of target clusters from a dataset.

## Results and discussion

A differential-character-finding algorithm was added to the InforBIO software and was tested with data for *Pseudomonas *strains to identify discriminative characteristics for *Pseudomonas *species with reference to phylogenetic clusters based on their 16S rRNA gene sequences.

### Identification of discriminative phenotypic characteristics for *Pseudomonas *species

#### Construction of the biologic database of *Pseudomonas *species

**Figure 2:**
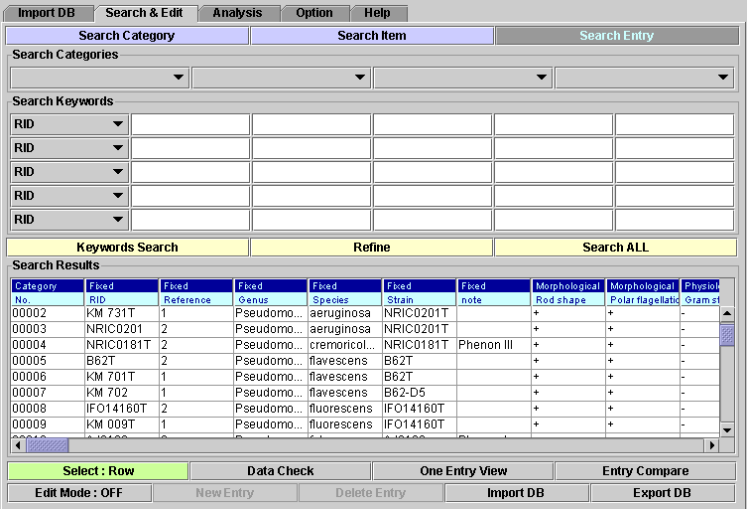
**InforBIO database screen**. The biologic database screen is shown. OTU data are recorded in a line.

Data, formatted with eXtensible Markup Language (XML) for the InforBIO software [see Additional file 1]. The phenotypic data of 36 OTUs comprising strains of *P. aeruginosa*, *P. cremoricolorata*, *P. flavescens*, *P. fluorescens*, *P. fulva*, *P. luteola*, *P. mendocina*, *P. oryzihabitans*, *P. parafulva*, *P. putida*, and *P. straminea *were obtained from published reports [18,19]. The dataset consisted of 144 phenotypic items to which two values for characterization (*n *= 2) were assigned [see Additional file 2], and the capture of the database screen in the InforBIO software is shown in figure 2.

#### Construction of a phylogenetic tree based on sequences from *Pseudomonas *species

**Figure 3:**
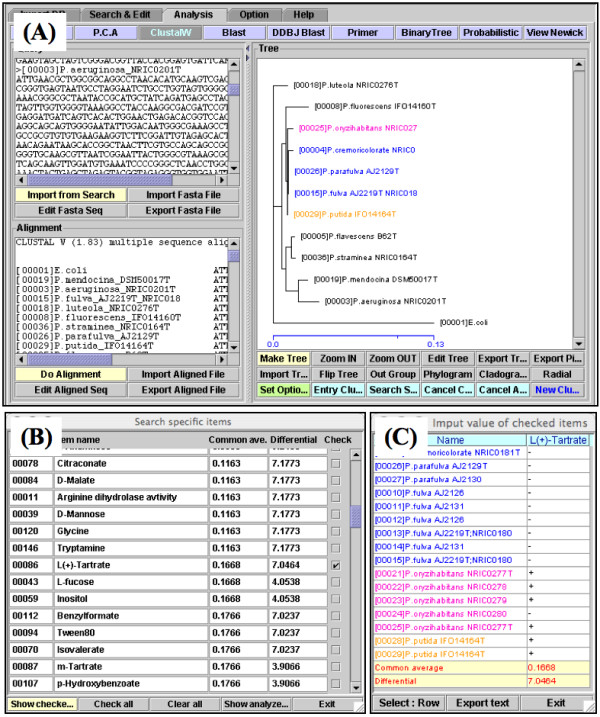
**Captures of result viewers of InforBIO**. (A) Result of phylogenetic analysis. Sequence data can be imported, edited, and aligned in the left window, and a tree is shown in the right window. Target clusters selected by clicking on the tree are shown in color. A scale bar indicates nucleotide substitution per position in the sequence. (B) Table of the results of the differential-character-finding analysis for clusters selected in (A). The common and differential scores of items are shown. (C) Table of the characteristics of OTUs of the item checked in (B).

We used the InforBIO software equipped with the DNAML program [14,15] to generate a phylogenetic tree based on 16S rRNA gene sequences of 11 *Pseudomonas *species after eliminating putative variable regions [20]. The phylogenetic tree constructed with the InforBIO software is shown in figure 3A and is supported by the past report [20].

#### Identification of discriminative characteristics for *Pseudomonas *species

**Figure 4:**
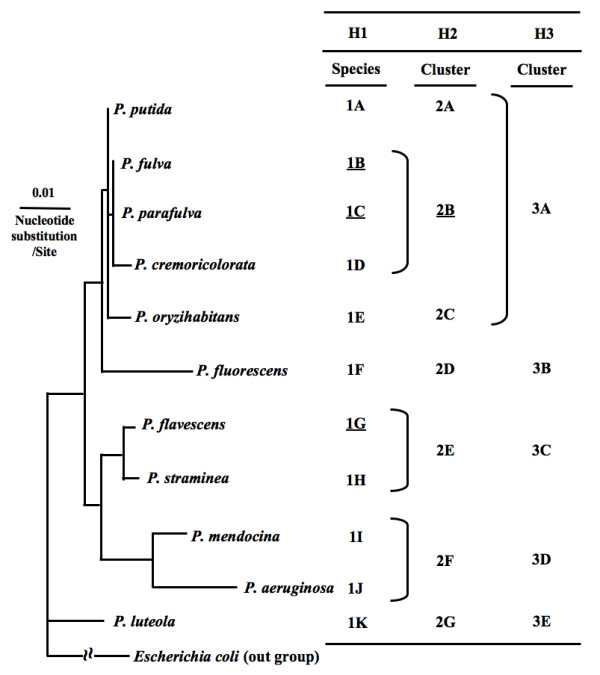
**Phylogenetic tree based on 16S rRNA gene sequences of *Pseudomonas *species**. Undiscriminated species and cluster in each step are underlined.

Species-discriminative characteristics for 11 species of *Pseudomonas *from the dataset were examined (*y *= 11, *a *= 0.95) by the algorithm. Thresholds were set as *C *≤ 0.1985 and *D *≥ 1.4474. As a result, 43 items were rejected because they did not satisfy the threshold condition of *C*_*thr *_(4 items) or *D*_*thr *_(39 items). In contrast, 14 items were identified as best discriminative, without exceptions, for *P. aeruginosa *(4 items), *P. cremoricolorata *(2 items), *P. fluorescens *(2 items), *P. mendocina *(1 item), and *P. putida *(5 items) Also, 5 best discriminative items, with few exceptions, for *P. luteola *(2 items), *P. oryzihabitans *(1 item), and *P. straminea *(2 items) were identified. However, no discriminative characteristics for *P. flavescens*, *P. fulva*, and *P. parafulva *were identified. Therefore, their discriminative characteristics were identified with reference to discriminative characteristics for clusters including the undiscriminated species on a phylogenetic tree based on 16S rRNA gene sequences of *Pseudomonas *species. In this study, a phylogenetic cluster including the undiscriminated species was analyzed hierarchically and stepwise from leaves to upper nodes (clusters) on a phylogenetic tree as shown in figure 4 until clusters with discriminative characteristics were detected. Hence, the undiscriminated species were discriminated by items that discriminate within each upper cluster.

As shown in figure 4, undiscriminated species were 1B, 1C, and 1G in the first step (H1) and were located in two clusters (2B and 2E) on the phylogenetic tree in the second step (H2). Discriminative characteristics for the two clusters were analyzed under the conditions described above. The 2E cluster is composed of *P. flavescens *and *P. straminea *and could be discriminated from six other clusters by a single characteristic, non-assimilation of L-isoleucine. Moreover, the two species could be discriminated from each other by 7 characteristics because their values for these items were opposite each other. Thus, at least two characteristics are needed for discrimination of these species from other *Pseudomonas *species. In contrast, no characteristics were identified that discriminated members of the 2B cluster composed of three species, including *P. fulva *and *P. parafulva*. Therefore, an upper cluster by the third step (H3), comprising five species from the addition of two species to the lower 2B cluster (3A by H3 in figure 4), was examined in the same manner and was discriminated by a single characteristic, non-reduction of nitrate. Additionally, best discriminative characteristics for *P. parafulva *(15 items) within the 3A cluster were identified, whereas those for *P. fulva *were not. To identify the discriminative characteristics for *P. fulva*, characteristics to discriminate the 2B cluster were identified within the 3A cluster, and the discriminative characteristic from the 2A and 2C clusters was non-assimilation of L-tartrate, whose *C *and *D *values were 0.1668 and 7.0464, respectively. Captures of the result screens of the InforBIO software are shown in figures 3B–3C. Finally, three best discriminative characteristics for *P. fulva *within the 2B cluster were identified. Therefore, at least three characteristics, non-reduction of nitrate, non-assimilation of L-tartrate, and one of the three discriminative characteristics for *P. fulva *within the 2B cluster, are needed to discriminate the species. Moreover, discriminative characteristics for upper clusters are effective as additional data for the discrimination of species, whose discriminative characteristics are tenuous. For instance, non-reduction of nitrate, a discriminative characteristic for the 3A cluster, is additional data for the discrimination of *P. oryzihabitans*, which is able to assimilate L-rhamnose. Consequently, the algorithm and the InforBIO software were effective for identification of characteristics that allowed discrimination of 11 *Pseudomonas *species with reference to discriminative characteristics for phylogenetic clusters. In addition, such an approach and results may be helpful to find the specific properties of species, which is important for phenotypic studies [21]. In the InforBIO software, the *C *and *D *thresholds cannot be set flexibly and have simply been set to 0.5 and 2, respectively. Thus, a table of detailed data of discriminative characteristics is provided by the InforBIO software as shown in figure 3C.

## Conclusion

We developed a differential-character-finding algorithm for the identification of the best characteristic to discriminate focused clusters. The algorithm can be used to analyze any type of cluster because it evaluates both intra-cluster and inter-cluster entropy. The common and differential scores are sensitive to taxon sampling. Thus, their thresholds are calculated with the number of defined clusters (*y*) and of values for an item (*n*) in addition to an acceptable frequency of the occurrence probability (*a*) of a value for an item. For continuous data, ranges can be set and converted into discrete data that can be analyzed by the algorithm in the InforBIO software. The set of ranges should include all possible values, but ranges should not overlap. These discrete ranges can then be regarded as values for the algorithm. There are algorithms available to identify sets of diagnostic keys [24] that can reduce a large dataset into compact, homogeneous data clusters. There are also tools for deterministic and probabilistic identification [25]. The unique feature of our differential-character-finding algorithm in the InforBIO software is that the system can identify the phenotypic characteristics that discriminate and are associated with the clusters of a phylogenetic tree. In current study, the phylogeny of protein-coding gene and protein sequences is analyzed in addition to 16S rRNA gene sequence phylogeny [26,27], and discriminative characteristics for clusters on a phylogenetic tree based on such sequences might be interesting for polyphasic analysis.

We demonstrated the algorithm in the InforBIO software with an actual dataset of *Pseudomonas *species. In the recent taxonomic studies of the genus *Pseudomonas*, specific characteristics of newly suggested species have been decided on the basis of the result from kits with many test items in addition to their phylogeny [26-28]. Also, the importance of species-discriminative phenotypic characteristics has become evident recently in taxonomic studies of species of other genera [29-33]. In this study, we successfully identified a set of phenotypic characteristics that were useful as diagnostic keys for *Pseudomonas *species. Discriminative characteristics for phylogenetic clusters as shown in the demonstration might be useful information for the finding of novel features for species. Therefore, the differential-character-finding algorithm and the InforBIO software are effective for identification of the characteristics that discriminate clusters from biologic data.

## Availability and requirements

Project name: InforBIO project;

Project homepage: ;

Operating systems: Windows 2000/XP, Macintosh OSX, Linux, UNIX;

Other requirements: CPU ≥ 800 MHz, Memory ≥ 256 MB, HD ≥ 50 MB, Screen resolution ≥ 800 × 600 pixels;

Programming language: Java (j2sdk1.4.2_05);

License: GNU GPL;

Any restrictions to use by non-academics: none.

## Authors' contributions

NT participated in the design and coordination of the study and drafted the manuscript. MU, SM, and HS conceived of the study and participated in its design. All authors read and approved the final manuscript.

## Supplementary Material

Additional file 1 This compressed file includes data files (such as Pseudomonas.xml) for InforBIO. Details of how to use the file are described in the file of ReadMe.ppt (Power Point file). Click here for file

Additional file 2 This file can be browsed by using PDF file viewer such as Acrobat Reader. Click here for file
