# Supplementary material for: Identification of discriminative characteristics for clusters from biologic data with InforBIO software
Source: BMC Bioinformatics. 2007 Aug 2;8:281. doi: 10.1186/1471-2105-8-281 (PMC1973088; doi:10.1186/1471-2105-8-281)
Supplement: Additional file 2 — This file can be browsed by using PDF file viewer such as Acrobat Reader. [file 1471-2105-8-281-S2.pdf]

Table S1. Dataset of phenotypic data for *Pseudomonas* strains

				Phenotypic characteristic																									
				Morphological		Physiological														Production of water-insoluble yellow pigment on		Production of water-soluble fluorescent pigment on			Production of pyocanin on		Growth at		
No.	*Pseudomonas* species	Strain*	Reference†	Rod shape	Polar flagellation	Gram stain	Motility	Arginine dihydrolase avtivity	Starch Hydrolysis	LEVAN formation from sucrose	Cleavage of aromatic compounds	Oxidation	Catalase activity	Oxidase activity	PHB accumulation	Casein hydrolysis	Nitrate reduction	Nitrate respiration	Growth on glutamate agr	Nutrientagar	TS agar	Pseudomonas agar F	King B	Kato and Ito's P1	Pseudomonas agar P	King A	pH3.6	4 degree C	41 degree C
[000002]	*Pseudomonas aeruginosa*	NRIC0201T	1	+	+	-	+	+	-	-	+	+	+	+	-	+	+	+	+	-	-	+	+	+	-	-	-	-	+
[000003]	*Pseudomonas aeruginosa*	NRIC0201T	2	+	+	-	+	+	-	-	+	+	+	+	-	+	+	+	+	-	-	+	+	+	-	+	-	-	+
[000004]	*Pseudomonas cremoricolorate*	NRIC0181T	2	+	+	-	+	-	-	-	-	+	+	+	-	-	-	-	+	+	+	-	-	-	-	-	-	+	-
[000005]	*Pseudomonas flavescens*	B62T	2	+	+	-	+	-	-	-	+	+	+	+	-	-	+	-	+	+	+	+	-	+	-	-	-	+	-
[000006]	*Pseudomonas flavescens*	B62T	1	+	+	-	+	-	-	-	+	+	+	+	-	+	+	-	+	+	+	+	-	+	-	-	-	+	-
[000007]	*Pseudomonas flavescens*	B62-D5	1	+	+	-	+	-	-	-	+	+	+	+	-	+	+	-	+	+	+	-	+	+	-	-	-	+	-
[000008]	*Pseudomonas fluorescens*	IFO14160T	2	+	+	-	+	+	-	+	+	+	+	+	-	-	+	-	+	-	-	+	+	+	-	-	-	+	-
[000009]	*Pseudomonas fluorescens*	IFO14160T	1	+	+	-	+	+	-	+	+	+	+	+	-	-	+	-	+	-	-	+	+	+	-	-	-	+	-
[000010]	*Pseudomonas fulva*	AJ2126	2	+	+	-	+	+	-	-	+	+	+	+	-	-	-	-	+	+	+	-	-	-	-	-	-	+	-
[000011]	*Pseudomonas fulva*	AJ2131	2	+	+	-	+	+	-	-	+	+	+	+	-	-	-	-	+	+	+	-	-	-	-	-	-	+	-
[000012]	*Pseudomonas fulva*	AJ2126	1	+	+	-	+	+	-	-	+	+	+	+	-	-	-	-	+	+	+	-	-	+	-	-	-	+	-
[000013]	*Pseudomonas fulva*	AJ2219T;NRIC0180	1	+	+	-	+	+	-	-	+	+	+	+	-	-	-	-	+	+	+	-	-	-	-	-	-	+	-
[000014]	*Pseudomonas fulva*	AJ2131	1	+	+	-	+	+	-	-	+	+	+	+	-	-	-	-	+	+	+	-	-	-	-	-	-	+	-
[000015]	*Pseudomonas fulva*	AJ2219T;NRIC0180	2	+	+	-	+	+	-	-	+	+	+	+	-	-	-	-	+	+	+	-	-	-	-	-	-	+	-
[000016]	*Pseudomonas luteola*	NRIC0276T	1	+	+	-	+	+	-	-	+	+	+	-	+	+	+	-	+	+	+	-	-	-	-	-	-	-	+
[000017]	*Pseudomonas luteola*	NRIC0275	1	+	+	-	+	+	-	-	+	+	+	-	+	+	+	-	+	+	+	-	-	-	-	-	-	-	+
[000018]	*Pseudomonas luteola*	NRIC0276T	2	+	+	-	+	+	-	-	+	+	+	-	+	+	+	-	+	+	+	-	-	-	-	-	-	-	+
[000019]	*Pseudomonas mendocina*	DSM50017T	3	+	+	-	+	+	-	-	+	+	+	+	-	-	+	+	+	-	-	-	-	-	-	-	-	-	+
[000020]	*Pseudomonas mendocina*	DSM50017T	1	+	+	-	+	+	-	-	+	+	+	+	-	-	+	+	+	-	-	-	-	-	-	-	-	-	+
[000021]	*Pseudomonas oryzihabitans*	NRIC0277T	1	+	+	-	+	-	-	-	+	+	+	-	+	+	-	-	+	+	+	-	-	-	-	-	-	-	-
[000022]	*Pseudomonas oryzihabitans*	NRIC0278	1	+	+	-	+	-	-	-	+	+	+	-	+	+	-	-	+	+	+	-	-	-	-	-	-	-	-
[000023]	*Pseudomonas oryzihabitans*	NRIC0279	1	+	+	-	+	-	-	-	+	+	+	-	+	+	-	-	+	+	+	-	-	-	-	-	-	+	-
[000024]	*Pseudomonas oryzihabitans*	NRIC0280	1	+	+	-	+	-	-	-	+	+	+	-	+	+	-	-	+	+	+	-	-	-	-	-	-	-	-
[000025]	*Pseudomonas oryzihabitans*	NRIC0277T	2	+	+	-	+	-	-	-	+	+	+	-	+	+	-	-	+	+	+	-	-	-	-	-	-	-	-
[000026]	*Pseudomonas parafulva*	AJ2129T	2	+	+	-	+	+	-	-	+	+	+	+	-	-	-	-	+	+	+	-	-	-	-	-	-	+	-
[000027]	*Pseudomonas parafulva*	AJ2130	2	+	+	-	+	+	-	-	+	+	+	+	-	-	-	-	+	+	+	-	-	-	-	-	-	-	-
[000028]	*Pseudomonas putida*	IFO14164T	1	+	+	-	+	+	-	-	+	+	+	+	-	-	-	-	+	-	-	-	+	-	-	-	-	+	-
[000029]	*Pseudomonas putida*	IFO14164T	2	+	+	-	+	+	-	-	+	+	+	+	-	-	-	-	+	-	-	-	+	+	-	-	-	+	-
[000030]	*Pseudomonas straminea*	NRIC0164T	1	+	+	-	+	-	-	-	+	+	+	+	-	+	+	-	+	+	+	-	-	-	-	-	-	+	-
[000031]	*Pseudomonas straminea*	AJ2124(T);IAM1598;CB-7	1	+	+	-	+	-	-	-	+	+	+	+	-	+	+	-	+	+	+	-	-	-	-	-	-	+	-
[000032]	*Pseudomonas straminea*	AJ2122	1	+	+	-	+	-	-	-	+	+	+	+	-	+	+	-	+	+	+	-	-	-	-	-	-	+	-
[000033]	*Pseudomonas straminea*	AJ2122-2	1	+	+	-	+	-	-	-	+	+	+	+	-	+	+	-	+	+	+	-	-	-	-	-	-	+	-
[000034]	*Pseudomonas straminea*	AJ2123-2	1	+	+	-	+	-	-	-	+	+	+	+	-	+	+	-	+	+	+	-	-	-	-	-	-	+	-
[000035]	*Pseudomonas straminea*	AJ2123	1	+	+	-	+	-	-	-	+	+	+	+	-	+	+	-	+	+	+	-	-	-	-	-	-	+	-
[000036]	*Pseudomonas straminea*	NRIC0164T	2	+	+	-	+	-	-	-	+	+	+	+	-	-	+	-	+	+	+	-	-	-	-	-	-	+	-
[000037]	*Pseudomonas straminea*	NRIC0182	2	+	+	-	+	-	-	-	+	+	+	+	-	-	+	-	+	+	+	-	-	-	-	-	-	+	-

*Abbreviations: AJ, Central Research Laboratories, Ajinomoto Co., Kawasaki, Japan; B, Obtained from D. J. Hildebrand, University of California, Berkeley, CA, USA; DSM, Deutsche Sammlung von Microorganismen und Zellkulturen, Braunschweig, Germany; IFO, Institute for Fermentation, Osaka, Japan; NRIC, NODAI Culture Collection, Tokyo University of Agriculture, Tokyo, Japan; T, Type strain.

† Referred from Uchino et al., 2000 (1), Uchino et al., 2001 (2) and unpublished data (3)

Table S1. (cont.)

				Phenotypic characteristic																								
				Assimilation of																								
No.	*Pseudomonas* species	Strain*	Reference†	meso-erythritol	D-Ribose	D-Arabinose	L-Arabinose	D-Xylose	D-Glucose	D-Mannose	D-Galactose	D-Fructose	L-Rhamnose	L-fucose	Sucrose	Maltose	Cellobiose	Trehalose	alpha-D-meribioses	Lactose	Raffinose	Solubel Starch	Inulin	Adonitol	D-Alabitol	L-Alabitol	D-xylitol	Sorbitol
[000002]	*Pseudomonas aeruginosa*	NRIC0201T	1	-	+	-	-	-	+	+	-	+	-	-	-	-	-	-	+	-	-	-	-	-	+	-	-	-
[000003]	*Pseudomonas aeruginosa*	NRIC0201T	2	+	+	-	-	-	+	-	-	+	-	-	-	-	-	-	-	-	-	-	-	-	+	-	-	-
[000004]	*Pseudomonas cremoricolorate*	NRIC0181T	2	-	-	-	-	-	+	-	-	+	-	-	-	-	-	-	-	-	-	-	-	-	-	-	-	-
[000005]	*Pseudomonas flavescens*	B62T	2	-	-	-	-	+	+	+	+	+	-	-	+	-	-	+	-	-	-	-	-	-	-	+	-	-
[000006]	*Pseudomonas flavescens*	B62T	1	-	-	-	-	+	+	+	+	+	-	-	+	-	-	+	-	-	-	-	-	-	-	+	-	-
[000007]	*Pseudomonas flavescens*	B62-D5	1	-	-	-	+	+	+	+	+	+	-	-	+	-	-	+	-	-	-	-	-	-	-	+	-	-
[000008]	*Pseudomonas fluorescens*	IFO14160T	2	+	+	-	+	+	+	+	+	+	-	-	+	-	-	+	-	-	-	-	-	+	+	+	+	+
[000009]	*Pseudomonas fluorescens*	IFO14160T	1	+	+	-	+	+	+	+	+	+	-	-	+	-	-	+	-	-	-	-	-	+	+	+	+	+
[000010]	*Pseudomonas fulva*	AJ2126	2	-	+	-	-	-	+	+	-	+	-	-	-	-	-	-	-	-	-	-	-	-	-	-	-	-
[000011]	*Pseudomonas fulva*	AJ2131	2	-	+	-	+	-	+	+	-	+	-	-	-	-	-	-	-	-	-	-	-	-	-	-	-	-
[000012]	*Pseudomonas fulva*	AJ2126	1	-	+	-	-	-	+	+	-	+	-	-	-	-	-	-	-	-	-	-	-	-	-	-	-	-
[000013]	*Pseudomonas fulva*	AJ2219T;NRIC0180	1	-	-	-	+	-	+	+	-	+	-	-	-	-	-	-	-	-	-	-	-	-	-	-	-	-
[000014]	*Pseudomonas fulva*	AJ2131	1	-	+	-	+	-	+	+	-	+	-	-	-	-	-	-	-	-	-	-	-	-	-	-	-	-
[000015]	*Pseudomonas fulva*	AJ2219T;NRIC0180	2	-	+	-	+	-	+	+	-	+	-	-	-	-	-	-	-	-	-	-	-	-	-	-	-	-
[000016]	*Pseudomonas luteola*	NRIC0276T	1	-	+	+	-	+	+	+	+	+	-	+	-	+	-	+	+	-	-	-	-	-	+	-	-	-
[000017]	*Pseudomonas luteola*	NRIC0275	1	-	+	+	+	+	+	+	+	+	+	+	+	+	-	+	+	-	-	-	-	-	+	-	-	-
[000018]	*Pseudomonas luteola*	NRIC0276T	2	-	+	-	+	+	+	+	+	+	-	+	-	+	-	+	+	-	-	-	-	-	+	-	-	-
[000019]	*Pseudomonas mendocina*	DSM50017T	3	-	-	-	-	-	+	-	-	+	-	-	-	-	-	-	-	-	-	-	-	-	-	+	-	-
[000020]	*Pseudomonas mendocina*	DSM50017T	1	-	-	-	-	-	+	-	-	+	-	-	-	-	-	-	-	-	-	-	-	-	-	+	-	-
[000021]	*Pseudomonas oryzihabitans*	NRIC0277T	1	-	+	+	+	+	+	+	+	+	+	+	-	+	-	+	+	-	-	-	-	-	+	-	-	+
[000022]	*Pseudomonas oryzihabitans*	NRIC0278	1	+	+	+	+	+	+	+	+	+	+	+	-	+	-	+	+	-	-	-	-	-	+	-	-	+
[000023]	*Pseudomonas oryzihabitans*	NRIC0279	1	+	+	+	+	+	+	+	+	+	+	+	-	+	-	+	-	-	-	-	-	-	+	-	-	+
[000024]	*Pseudomonas oryzihabitans*	NRIC0280	1	-	+	-	+	+	+	+	+	+	+	-	-	+	-	+	-	-	-	-	-	-	+	-	-	+
[000025]	*Pseudomonas oryzihabitans*	NRIC0277T	2	-	+	+	+	+	+	+	+	+	+	+	-	+	-	+	+	-	-	-	-	-	+	-	-	+
[000026]	*Pseudomonas parafulva*	AJ2129T	2	-	+	+	-	-	+	+	-	+	-	-	-	-	-	-	-	-	-	-	-	+	-	-	-	-
[000027]	*Pseudomonas parafulva*	AJ2130	2	-	+	-	-	-	+	+	-	+	-	-	-	-	-	-	-	-	-	-	-	+	-	-	-	-
[000028]	*Pseudomonas putida*	IFO14164T	1	-	+	-	+	-	+	-	-	+	-	-	+	-	-	-	-	-	-	-	-	-	-	-	-	-
[000029]	*Pseudomonas putida*	IFO14164T	2	-	+	-	+	-	+	-	-	+	-	-	+	-	-	-	-	-	-	-	-	-	-	-	-	-
[000030]	*Pseudomonas straminea*	NRIC0164T	1	-	+	-	+	+	+	+	+	+	-	-	-	-	-	-	-	-	+	-	-	-	-	+	-	-
[000031]	*Pseudomonas straminea*	AJ2124(T);IAM1598;CB-7	1	-	-	-	+	+	+	+	+	+	-	-	-	-	-	-	-	-	-	-	-	-	+	-	-	-
[000032]	*Pseudomonas straminea*	AJ2122	1	-	-	-	+	+	+	+	+	+	-	-	-	-	-	-	-	-	-	-	-	-	-	-	-	-
[000033]	*Pseudomonas straminea*	AJ2122-2	1	-	+	-	+	+	+	+	+	+	-	-	-	-	-	-	-	-	-	-	-	-	+	-	-	-
[000034]	*Pseudomonas straminea*	AJ2123-2	1	-	-	-	+	+	+	+	+	+	-	-	-	-	-	-	-	-	-	-	-	-	+	-	-	-
[000035]	*Pseudomonas straminea*	AJ2123	1	-	-	-	+	+	+	+	+	+	-	-	-	-	-	-	-	-	-	-	-	-	+	-	-	-
[000036]	*Pseudomonas straminea*	NRIC0164T	2	-	+	-	+	+	+	+	+	+	-	-	-	-	-	-	-	-	+	-	-	-	-	+	-	-
[000037]	*Pseudomonas straminea*	NRIC0182	2	-	-	-	-	-	+	+	+	+	-	-	-	-	-	-	-	-	-	-	-	-	+	-	-	-

*Abbreviations: AJ, Central Research Laboratories, Ajinomoto Co., Kawasaki, Japan; B, Obtained from D. J. Hildebrand, University of California, Berkeley, CA, USA; DSM, Deutsche Sammlung von Microorganismen und Zellkulturen, Braunschweig, Germany; IFO, Institute for Fermentation, Osaka, Japan; NRIC, NODAI Culture Collection, Tokyo University of Agriculture, Tokyo, Japan; T, Type strain.

† Referred from Uchino et al., 2000 (1), Uchino et al., 2001 (2) and unpublished data (3)

Table S1. (cont.)

No.	*Pseudomonas* species	Strain*	Reference†	Mannitol	Inositol	Gluconate	2-Ketogluconate	5-ketogluconate	Glucosamin	Acetate	Propionate	Butyrate	Valerate	Caprate	Isobutyrate	Isovalerate	Malonate	Succinate	Adipate	Fumalate	Azelate	Sebacate	Itaconate	Citraconate	Mucate	Glycerate	L-Lactate	Hydroxybutyrate
				Phenotypic characteristic: Assimilation of																								
[000002]	*Pseudomonas aeruginosa*	NRIC0201T	1	+	-	+	+	+	-	-	+	+	+	+	-	+	+	+	+	+	+	+	+	-	-	-	-	+
[000003]	*Pseudomonas aeruginosa*	NRIC0201T	2	+	-	+	+	+	-	+	+	+	+	+	+	+	+	-	+	+	+	+	+	-	-	-	+	+
[000004]	*Pseudomonas cremoricolorate*	NRIC0181T	2	-	-	+	-	-	-	+	+	-	-	-	-	-	+	+	-	+	-	-	-	+	+	-	+	-
[000005]	*Pseudomonas flavescens*	B62T	2	+	+	+	-	+	-	+	-	-	+	+	-	-	+	+	-	+	-	-	+	-	+	-	+	-
[000006]	*Pseudomonas flavescens*	B62T	1	+	+	+	-	+	-	+	-	-	+	+	-	-	+	+	-	+	-	-	+	-	+	-	+	-
[000007]	*Pseudomonas flavescens*	B62-D5	1	+	-	+	-	+	-	+	-	-	+	+	-	-	+	+	-	+	-	-	+	-	+	-	+	-
[000008]	*Pseudomonas fluorescens*	IFO14160T	2	+	+	+	-	+	+	+	+	+	+	+	+	-	+	+	-	+	-	-	+	+	+	-	+	+
[000009]	*Pseudomonas fluorescens*	IFO14160T	1	+	+	+	-	+	+	+	+	+	+	+	+	-	+	+	-	+	-	-	+	+	+	-	+	+
[000010]	*Pseudomonas fulva*	AJ2126	2	-	-	+	+	-	-	+	+	-	-	+	-	+	-	+	-	+	-	-	+	-	+	+	+	-
[000011]	*Pseudomonas fulva*	AJ2131	2	-	-	+	+	-	-	+	+	-	-	+	-	+	-	+	-	+	-	-	+	-	+	+	+	-
[000012]	*Pseudomonas fulva*	AJ2126	1	-	-	+	+	-	-	+	+	-	-	+	-	-	+	+	-	+	-	-	+	-	+	+	+	-
[000013]	*Pseudomonas fulva*	AJ2219T;NRIC0180	1	-	-	+	+	-	-	+	+	+	+	+	+	+	+	+	-	+	-	-	+	-	+	+	+	-
[000014]	*Pseudomonas fulva*	AJ2131	1	-	-	+	+	-	-	+	+	-	-	+	-	+	-	+	-	+	-	-	+	-	+	+	+	-
[000015]	*Pseudomonas fulva*	AJ2219T;NRIC0180	2	-	-	+	+	-	-	+	+	-	-	+	+	+	-	+	-	+	-	-	+	-	+	+	+	-
[000016]	*Pseudomonas luteola*	NRIC0276T	1	+	+	+	+	+	+	+	+	+	-	+	+	-	-	+	-	+	-	-	-	-	+	-	+	+
[000017]	*Pseudomonas luteola*	NRIC0275	1	+	+	+	+	+	+	-	+	+	-	+	+	-	-	+	-	+	-	-	-	-	+	-	+	-
[000018]	*Pseudomonas luteola*	NRIC0276T	2	+	+	+	+	+	+	+	+	+	-	+	+	-	-	+	-	+	-	-	-	-	+	-	+	+
[000019]	*Pseudomonas mendocina*	DSM50017T	3	-	-	+	-	-	-	-	+	-	-	+	-	-	-	+	-	+	-	-	+	-	-	-	+	+
[000020]	*Pseudomonas mendocina*	DSM50017T	1	-	-	+	-	-	-	-	+	-	-	+	-	-	-	+	-	+	-	-	+	-	-	-	+	+
[000021]	*Pseudomonas oryzihabitans*	NRIC0277T	1	+	+	+	+	+	-	+	+	-	-	+	-	-	+	+	-	+	-	-	+	+	+	+	+	-
[000022]	*Pseudomonas oryzihabitans*	NRIC0278	1	+	+	+	+	+	+	+	+	-	-	+	-	-	+	+	-	+	-	-	+	+	+	+	+	-
[000023]	*Pseudomonas oryzihabitans*	NRIC0279	1	+	+	+	+	+	+	+	+	-	-	+	-	-	+	+	-	+	-	-	+	+	+	-	+	-
[000024]	*Pseudomonas oryzihabitans*	NRIC0280	1	+	-	+	+	+	+	+	+	-	-	+	+	-	-	+	-	+	-	-	+	+	+	+	+	-
[000025]	*Pseudomonas oryzihabitans*	NRIC0277T	2	+	+	+	+	+	-	+	+	-	-	+	-	-	+	+	-	+	-	-	+	+	+	+	+	-
[000026]	*Pseudomonas parafulva*	AJ2129T	2	-	-	+	+	-	-	+	+	+	+	+	+	+	+	+	-	+	-	-	-	-	+	+	+	+
[000027]	*Pseudomonas parafulva*	AJ2130	2	-	-	+	+	-	+	+	+	+	+	+	+	+	-	-	-	+	-	-	-	-	+	+	+	-
[000028]	*Pseudomonas putida*	IFO14164T	1	-	-	+	+	-	-	+	+	+	+	+	+	+	+	+	-	+	-	-	+	-	+	+	+	-
[000029]	*Pseudomonas putida*	IFO14164T	2	-	-	+	+	-	-	+	+	+	+	+	+	+	+	+	-	+	-	-	+	-	+	+	+	-
[000030]	*Pseudomonas straminea*	NRIC0164T	1	+	-	+	-	-	-	-	-	-	-	+	-	-	-	+	-	+	-	-	+	-	+	-	+	+
[000031]	*Pseudomonas straminea*	AJ2124(T);IAM1598;CB-7	1	+	-	+	-	-	-	-	-	-	-	+	-	-	-	+	-	+	-	-	+	-	+	-	+	+
[000032]	*Pseudomonas straminea*	AJ2122	1	+	-	+	-	-	-	-	-	-	-	+	-	-	-	+	-	+	-	-	+	-	+	-	+	+
[000033]	*Pseudomonas straminea*	AJ2122-2	1	+	-	+	-	-	-	+	+	-	-	+	-	-	-	+	-	-	-	-	+	-	+	-	+	-
[000034]	*Pseudomonas straminea*	AJ2123-2	1	+	-	+	-	-	-	-	-	-	-	+	-	-	+	+	-	+	-	-	+	-	+	-	+	+
[000035]	*Pseudomonas straminea*	AJ2123	1	+	-	+	-	-	-	+	+	-	-	+	-	-	+	+	-	+	-	-	+	-	+	+	+	+
[000036]	*Pseudomonas straminea*	NRIC0164T	2	+	-	+	-	-	-	-	-	-	-	+	-	-	-	+	-	+	-	-	+	-	+	-	+	+
[000037]	*Pseudomonas straminea*	NRIC0182	2	-	+	+	-	-	-	-	-	-	-	+	-	-	+	+	-	+	-	-	+	-	+	-	+	+

*Abbreviations: AJ, Central Research Laboratories, Ajinomoto Co., Kawasaki, Japan; B, Obtained from D. J. Hildebrand, University of California, Berkeley, CA, USA; DSM, Deutsche Sammlung von Microorganismen und Zellkulturen, Braunschweig, Germany; IFO, Institute for Fermentation, Osaka, Japan; NRIC, NODAI Culture Collection, Tokyo University of Agriculture, Tokyo, Japan; T, Type strain.

† Referred from Uchino et al., 2000 (1), Uchino et al., 2001 (2) and unpublished data (3)

Table S1. (cont.)

No.	*Pseudomonas* species	Strain*	Reference†	Phenotypic characteristic: Assimilation of D-Malate	D(-)-Tartrate	L(+)-Tartrate	m-Tartrate	Citrate	D-Galactonolactone	Pyruvate	Levulinate	DL-2-Aminobutyrate	PHB	Tween80	Ethanol	n-Propanol	n-Butanol	isobutanol	1-Hexanol	Geraniol	Ethyleneglycol	Propyleneglycol	Phenol	Benzoate	m-Hydroxybenzoate	p-Hydroxybenzoate	Protocatecuate	Gentisate
[000002]	*Pseudomonas aeruginosa*	NRIC0201T	1	-	-	-	-	+	+	-	-	-	-	-	+	+	-	+	-	+	-	+	-	+	-	+	-	+
[000003]	*Pseudomonas aeruginosa*	NRIC0201T	2	-	-	-	-	+	-	+	+	-	-	-	+	+	+	+	-	+	-	+	-	+	-	+	-	-
[000004]	*Pseudomonas cremoricolorate*	NRIC0181T	2	+	-	-	-	+	-	+	-	-	-	+	+	+	-	-	-	-	-	+	-	-	-	-	-	-
[000005]	*Pseudomonas flavescens*	B62T	2	-	-	-	+	+	+	+	+	-	-	-	-	-	-	-	-	-	-	-	-	-	+	+	-	-
[000006]	*Pseudomonas flavescens*	B62T	1	-	-	-	+	+	+	+	+	-	-	-	-	-	-	-	-	-	-	-	-	-	+	+	-	-
[000007]	*Pseudomonas flavescens*	B62-D5	1	-	-	-	+	+	+	+	+	-	-	+	-	-	-	-	-	-	-	-	-	-	+	+	-	-
[000008]	*Pseudomonas fluorescens*	IFO14160T	2	-	-	-	+	+	+	+	-	-	-	-	-	-	-	-	-	-	-	-	-	-	+	+	-	-
[000009]	*Pseudomonas fluorescens*	IFO14160T	1	-	-	-	+	+	+	+	-	-	-	-	-	-	-	-	-	-	-	-	-	-	+	+	-	-
[000010]	*Pseudomonas fulva*	AJ2126	2	+	-	-	+	+	+	+	+	-	-	+	+	+	-	-	-	-	-	+	-	-	-	-	-	-
[000011]	*Pseudomonas fulva*	AJ2131	2	+	-	-	-	+	+	+	+	-	-	+	+	+	-	-	+	-	-	+	-	-	-	-	-	-
[000012]	*Pseudomonas fulva*	AJ2126	1	+	-	-	+	+	-	+	+	-	-	+	-	+	-	-	-	-	-	+	-	-	-	-	-	-
[000013]	*Pseudomonas fulva*	AJ2219T;NRIC0180	1	-	-	-	-	+	-	+	-	-	-	+	+	+	-	+	-	-	-	+	-	-	-	-	-	-
[000014]	*Pseudomonas fulva*	AJ2131	1	+	-	-	-	+	+	+	+	-	-	+	+	+	-	-	+	-	-	+	-	-	-	-	-	-
[000015]	*Pseudomonas fulva*	AJ2219T;NRIC0180	2	+	-	-	-	+	-	+	+	-	-	+	+	+	-	+	+	-	-	+	-	-	-	-	-	-
[000016]	*Pseudomonas luteola*	NRIC0276T	1	+	-	-	+	+	+	+	-	-	-	+	+	+	+	+	+	-	-	+	-	-	-	+	+	-
[000017]	*Pseudomonas luteola*	NRIC0275	1	+	-	-	+	+	+	+	-	-	-	+	+	+	+	-	+	+	-	+	-	-	-	+	+	-
[000018]	*Pseudomonas luteola*	NRIC0276T	2	+	-	-	+	+	+	+	-	-	-	+	+	+	+	+	+	-	-	+	-	-	-	+	+	-
[000019]	*Pseudomonas mendocina*	DSM50017T	3	+	-	-	-	+	-	+	-	-	-	-	+	+	+	-	+	+	+	+	-	+	-	-	-	-
[000020]	*Pseudomonas mendocina*	DSM50017T	1	+	-	-	-	+	-	+	-	-	-	-	+	+	+	-	+	+	+	+	-	+	-	-	-	-
[000021]	*Pseudomonas oryzihabitans*	NRIC0277T	1	+	-	+	+	+	+	+	-	-	-	+	+	+	-	+	+	-	-	-	-	-	-	+	-	-
[000022]	*Pseudomonas oryzihabitans*	NRIC0278	1	+	+	+	+	+	+	+	-	-	-	+	+	+	-	+	-	-	-	-	-	-	-	+	+	-
[000023]	*Pseudomonas oryzihabitans*	NRIC0279	1	+	-	+	+	+	+	+	-	-	-	+	+	+	-	-	-	-	-	-	-	-	-	+	+	-
[000024]	*Pseudomonas oryzihabitans*	NRIC0280	1	+	-	-	+	+	+	+	-	-	-	+	+	+	-	-	+	-	-	-	-	-	-	+	+	-
[000025]	*Pseudomonas oryzihabitans*	NRIC0277T	2	+	-	+	+	+	+	+	-	-	-	+	+	+	-	+	+	-	-	-	-	-	-	+	-	-
[000026]	*Pseudomonas parafulva*	AJ2129T	2	+	-	-	-	+	-	+	-	-	-	-	+	+	-	+	+	-	-	+	-	-	-	+	-	-
[000027]	*Pseudomonas parafulva*	AJ2130	2	+	-	-	-	+	-	+	-	-	-	-	+	+	-	+	+	-	-	+	-	-	-	+	-	-
[000028]	*Pseudomonas putida*	IFO14164T	1	-	-	+	+	+	-	+	+	+	-	-	+	+	+	-	+	-	-	+	-	+	+	+	-	-
[000029]	*Pseudomonas putida*	IFO14164T	2	-	-	+	+	+	-	+	+	+	-	-	+	+	+	-	+	-	-	+	-	+	+	+	-	-
[000030]	*Pseudomonas straminea*	NRIC0164T	1	-	-	-	-	+	+	+	-	-	-	-	-	-	-	-	-	-	-	-	-	+	-	+	-	-
[000031]	*Pseudomonas straminea*	AJ2124(T);IAM1598;CB-7	1	-	-	-	-	+	+	+	-	-	-	-	-	-	-	-	-	-	-	-	-	+	-	+	-	-
[000032]	*Pseudomonas straminea*	AJ2122	1	-	-	-	-	+	-	+	-	-	-	-	-	-	-	-	-	-	-	-	-	+	-	+	-	-
[000033]	*Pseudomonas straminea*	AJ2122-2	1	-	-	-	-	+	+	-	-	-	-	-	-	-	-	-	-	-	-	-	-	+	-	+	-	-
[000034]	*Pseudomonas straminea*	AJ2123-2	1	-	-	-	-	+	-	+	-	-	-	-	+	-	-	-	-	-	-	-	-	+	-	+	-	-
[000035]	*Pseudomonas straminea*	AJ2123	1	-	-	-	-	+	+	+	-	-	-	-	-	-	-	-	-	-	-	-	-	+	-	+	-	-
[000036]	*Pseudomonas straminea*	NRIC0164T	2	-	-	-	-	+	+	+	-	-	-	-	-	-	-	-	-	-	-	-	-	+	-	+	-	-
[000037]	*Pseudomonas straminea*	NRIC0182	2	-	-	-	+	+	+	+	-	-	-	-	-	-	-	-	-	-	-	-	-	-	-	+	-	-

*Abbreviations: AJ, Central Research Laboratories, Ajinomoto Co., Kawasaki, Japan; B, Obtained from D. J. Hildebrand, University of California, Berkeley, CA, USA; DSM, Deutsche Sammlung von Microorganismen und Zellkulturen, Braunschweig, Germany; IFO, Institute for Fermentation, Osaka, Japan; NRIC, NODAI Culture Collection, Tokyo University of Agriculture, Tokyo, Japan; T, Type strain.

† Referred from Uchino et al., 2000 (1), Uchino et al., 2001 (2) and unpublished data (3)

Table S1. (cont.)

No.	*Pseudomonas* species	Strain*	Reference†	D-Mandelate	L-Mandelate	Benzylformate	Xylene	Phenylacetate	p-Aminobenzoate	Naphthalene	inosine	Taurocholate	Testosterone	Glycine	beta-Alanine	L-Valine	L-Leusine	L-Isoleusine	D-Aspartate	L-Serine	L-Threonine	L-Phenylalanine	L-Tryptophan	D-Tryptophan	L-Lysine	L-Ornithine	L-Histidine	DL-Norvaline
				Phenotypic characteristic: Assimilation of																								
[000002]	*Pseudomonas aeruginosa*	NRIC0201T	1	-	+	+	-	-	-	-	+	-	-	+	+	+	+	+	-	+	-	+	+	-	+	+	-	+
[000003]	*Pseudomonas aeruginosa*	NRIC0201T	2	-	+	+	-	-	-	-	+	-	-	+	+	+	+	+	-	+	-	+	+	-	+	+	+	+
[000004]	*Pseudomonas cremoricolorate*	NRIC0181T	2	-	-	-	-	-	-	-	-	-	-	-	+	+	+	+	-	+	-	-	-	-	+	-	-	-
[000005]	*Pseudomonas flavescens*	B62T	2	-	-	-	-	-	-	-	+	-	-	-	-	-	+	-	-	+	-	-	-	-	-	-	+	-
[000006]	*Pseudomonas flavescens*	B62T	1	-	-	-	-	-	-	-	+	-	-	-	+	-	+	-	-	+	-	-	-	-	-	-	+	-
[000007]	*Pseudomonas flavescens*	B62-D5	1	-	-	-	-	-	-	-	+	-	-	+	+	-	+	-	-	+	-	-	-	-	-	-	+	-
[000008]	*Pseudomonas fluorescens*	IFO14160T	2	-	-	-	-	-	-	-	+	-	-	-	+	+	+	+	-	+	-	+	+	-	-	+	+	-
[000009]	*Pseudomonas fluorescens*	IFO14160T	1	-	-	-	-	-	-	-	+	-	-	-	+	+	+	+	-	+	-	+	+	-	-	+	+	-
[000010]	*Pseudomonas fulva*	AJ2126	2	-	-	-	-	-	-	-	+	-	-	+	+	+	+	+	-	+	-	-	-	-	+	+	+	-
[000011]	*Pseudomonas fulva*	AJ2131	2	-	-	+	-	+	-	-	+	-	-	+	+	+	+	+	-	+	+	+	-	-	+	+	-	-
[000012]	*Pseudomonas fulva*	AJ2126	1	-	-	-	-	-	-	-	+	-	-	+	+	+	+	+	-	+	-	-	-	-	+	+	+	-
[000013]	*Pseudomonas fulva*	AJ2219T;NRIC0180	1	-	-	-	-	+	-	-	+	-	-	+	+	+	+	+	-	+	+	+	-	-	+	+	+	-
[000014]	*Pseudomonas fulva*	AJ2131	1	-	-	+	-	+	-	-	+	-	-	+	+	+	+	+	-	+	+	+	-	-	+	+	-	-
[000015]	*Pseudomonas fulva*	AJ2219T;NRIC0180	2	-	-	-	-	+	-	-	+	-	-	+	+	+	+	+	-	+	+	+	-	-	+	+	+	-
[000016]	*Pseudomonas luteola*	NRIC0276T	1	-	-	-	-	-	-	-	+	-	-	-	+	-	+	+	-	+	-	-	-	-	+	+	+	-
[000017]	*Pseudomonas luteola*	NRIC0275	1	-	-	-	-	-	-	-	+	+	-	-	+	-	-	+	-	+	-	-	-	-	-	+	-	-
[000018]	*Pseudomonas luteola*	NRIC0276T	2	-	-	-	-	-	-	-	+	-	-	-	+	-	+	+	-	+	-	-	-	-	+	+	+	-
[000019]	*Pseudomonas mendocina*	DSM50017T	3	-	-	-	-	-	-	-	-	-	-	+	+	+	+	+	-	+	+	+	-	-	+	+	+	+
[000020]	*Pseudomonas mendocina*	DSM50017T	1	-	-	-	-	-	-	-	-	-	-	+	+	+	+	+	-	+	+	+	-	-	+	+	+	+
[000021]	*Pseudomonas oryzihabitans*	NRIC0277T	1	-	-	-	-	-	-	-	+	-	-	-	+	+	+	+	-	+	-	-	-	-	-	-	-	-
[000022]	*Pseudomonas oryzihabitans*	NRIC0278	1	-	-	-	-	-	-	-	+	-	-	-	-	+	+	+	-	+	-	-	-	-	-	+	+	-
[000023]	*Pseudomonas oryzihabitans*	NRIC0279	1	-	-	-	-	-	-	-	+	-	-	-	-	+	-	+	-	+	-	-	-	-	+	-	+	-
[000024]	*Pseudomonas oryzihabitans*	NRIC0280	1	-	-	-	-	-	-	-	-	-	-	-	-	+	-	+	-	+	-	-	-	-	+	-	+	-
[000025]	*Pseudomonas oryzihabitans*	NRIC0277T	2	-	-	-	-	-	-	-	+	-	-	-	+	+	+	+	-	+	-	-	-	-	-	-	-	-
[000026]	*Pseudomonas parafulva*	AJ2129T	2	-	-	-	-	-	-	-	+	-	-	+	+	+	+	+	-	+	-	+	-	-	+	+	+	-
[000027]	*Pseudomonas parafulva*	AJ2130	2	-	-	-	-	-	-	-	+	-	-	+	+	+	+	+	-	+	-	+	-	-	+	+	-	-
[000028]	*Pseudomonas putida*	IFO14164T	1	+	+	+	-	+	-	-	+	+	-	+	+	+	+	+	-	+	+	+	-	-	+	+	+	+
[000029]	*Pseudomonas putida*	IFO14164T	2	+	+	+	-	+	-	-	+	+	-	+	+	+	+	+	-	+	+	+	-	-	+	+	+	+
[000030]	*Pseudomonas straminea*	NRIC0164T	1	-	-	-	-	-	-	-	-	-	-	-	-	-	-	-	-	+	-	-	-	-	-	-	+	-
[000031]	*Pseudomonas straminea*	AJ2124(T);IAM1598;CB-7	1	-	-	-	-	-	-	-	+	-	-	+	-	-	-	-	-	+	-	-	-	-	-	-	+	-
[000032]	*Pseudomonas straminea*	AJ2122	1	-	-	+	-	-	-	-	-	-	-	-	-	-	-	-	-	+	-	-	-	-	-	-	+	-
[000033]	*Pseudomonas straminea*	AJ2122-2	1	-	-	-	-	-	-	-	-	-	-	-	-	-	-	-	-	+	-	-	-	-	-	-	-	-
[000034]	*Pseudomonas straminea*	AJ2123-2	1	-	-	-	-	-	-	-	-	-	-	-	-	-	-	-	-	+	-	-	-	-	-	-	-	-
[000035]	*Pseudomonas straminea*	AJ2123	1	-	-	-	-	-	-	-	-	-	-	-	-	-	-	-	-	+	-	-	-	-	-	-	+	-
[000036]	*Pseudomonas straminea*	NRIC0164T	2	-	-	-	-	-	-	-	-	-	-	-	-	-	-	-	-	+	-	-	-	-	-	-	+	-
[000037]	*Pseudomonas straminea*	NRIC0182	2	-	-	+	-	-	-	-	-	-	-	-	-	-	-	-	-	-	-	-	-	-	-	-	-	-

*Abbreviations: AJ, Central Research Laboratories, Ajinomoto Co., Kawasaki, Japan; B, Obtained from D. J. Hildebrand, University of California, Berkeley, CA, USA; DSM, Deutsche Sammlung von Microorganismen und Zellkulturen, Braunschweig, Germany; IFO, Institute for Fermentation, Osaka, Japan; NRIC, NODAI Culture Collection, Tokyo University of Agriculture, Tokyo, Japan; T, Type strain.

† Referred from Uchino et al., 2000 (1), Uchino et al., 2001 (2) and unpublished data (3)

Table S1. (cont.)

No.	*Pseudomonas* species	Strain*	Reference†	Phenotypic characteristic: Assimilation of														
				Creatine	Anthranilate	L-Citrulline	Hippurate	Pantothenate	Nicotinate	Glutathione	Trigonelline	Acetamide	Butylamine	Tryptamine	Amylamine	Benzylamine	Betaine	Dodecane
[000002]	*Pseudomonas aeruginosa*	NRIC0201T	1	-	-	+	-	+	-	-	-	+	-	-	-	-	+	-
[000003]	*Pseudomonas aeruginosa*	NRIC0201T	2	-	+	-	-	-	-	-	-	+	-	-	-	+	+	-
[000004]	*Pseudomonas cremoricolorate*	NRIC0181T	2	-	-	-	-	-	-	-	+	-	+	-	+	-	+	-
[000005]	*Pseudomonas flavescens*	B62T	2	-	-	+	-	-	-	-	-	-	-	-	+	-	+	-
[000006]	*Pseudomonas flavescens*	B62T	1	-	-	+	-	-	-	-	-	-	-	-	+	-	+	-
[000007]	*Pseudomonas flavescens*	B62-D5	1	-	-	+	-	-	-	-	-	-	-	+	+	-	+	-
[000008]	*Pseudomonas fluorescens*	IFO14160T	2	-	-	-	-	-	+	-	-	-	-	-	-	-	+	-
[000009]	*Pseudomonas fluorescens*	IFO14160T	1	-	-	-	-	-	+	-	-	-	-	-	-	-	+	-
[000010]	*Pseudomonas fulva*	AJ2126	2	-	-	+	-	-	+	-	+	-	+	-	+	-	+	-
[000011]	*Pseudomonas fulva*	AJ2131	2	-	-	+	-	-	+	-	+	-	+	-	-	-	+	-
[000012]	*Pseudomonas fulva*	AJ2126	1	-	-	+	-	-	+	-	+	-	+	-	-	-	+	-
[000013]	*Pseudomonas fulva*	AJ2219T;NRIC0180	1	-	-	+	-	-	+	-	+	-	+	-	-	-	+	-
[000014]	*Pseudomonas fulva*	AJ2131	1	-	-	+	-	-	+	-	+	-	+	-	-	-	+	+
[000015]	*Pseudomonas fulva*	AJ2219T;NRIC0180	2	-	-	+	-	-	+	-	+	-	+	-	-	-	+	-
[000016]	*Pseudomonas luteola*	NRIC0276T	1	-	-	-	-	+	-	-	-	-	-	-	-	-	+	-
[000017]	*Pseudomonas luteola*	NRIC0275	1	-	-	-	-	+	-	-	+	-	-	-	-	-	+	-
[000018]	*Pseudomonas luteola*	NRIC0276T	2	-	-	-	-	+	-	-	-	-	-	-	-	-	+	-
[000019]	*Pseudomonas mendocina*	DSM50017T	3	-	-	+	-	-	-	-	-	-	-	-	-	-	+	-
[000020]	*Pseudomonas mendocina*	DSM50017T	1	-	-	+	-	-	-	-	-	-	-	-	-	-	+	-
[000021]	*Pseudomonas oryzihabitans*	NRIC0277T	1	-	-	-	-	-	-	-	-	-	-	-	-	-	+	-
[000022]	*Pseudomonas oryzihabitans*	NRIC0278	1	-	-	-	-	-	-	+	+	-	-	-	-	-	+	-
[000023]	*Pseudomonas oryzihabitans*	NRIC0279	1	-	-	-	-	-	-	-	+	-	-	-	-	-	+	-
[000024]	*Pseudomonas oryzihabitans*	NRIC0280	1	-	-	-	-	+	-	-	+	-	-	-	-	-	+	-
[000025]	*Pseudomonas oryzihabitans*	NRIC0277T	2	-	-	-	-	-	-	-	-	-	-	-	-	-	+	-
[000026]	*Pseudomonas parafulva*	AJ2129T	2	-	-	-	-	-	-	-	+	-	+	+	+	-	+	-
[000027]	*Pseudomonas parafulva*	AJ2130	2	-	-	-	-	-	-	-	+	-	+	-	+	-	+	-
[000028]	*Pseudomonas putida*	IFO14164T	1	+	-	+	+	-	+	-	+	-	+	+	+	+	+	-
[000029]	*Pseudomonas putida*	IFO14164T	2	+	-	+	+	-	+	-	+	-	+	+	+	+	+	-
[000030]	*Pseudomonas straminea*	NRIC0164T	1	-	-	-	-	-	+	-	+	-	-	-	-	-	+	-
[000031]	*Pseudomonas straminea*	AJ2124(T);IAM1598;CB-7	1	-	-	-	-	-	+	-	+	-	-	-	-	-	+	-
[000032]	*Pseudomonas straminea*	AJ2122	1	-	-	-	-	-	+	-	-	-	-	+	-	+	+	-
[000033]	*Pseudomonas straminea*	AJ2122-2	1	-	-	-	-	-	+	-	-	-	-	+	-	+	+	-
[000034]	*Pseudomonas straminea*	AJ2123-2	1	-	-	-	-	-	+	-	+	-	-	-	-	-	+	-
[000035]	*Pseudomonas straminea*	AJ2123	1	-	-	-	-	-	+	-	+	-	-	-	+	-	+	-
[000036]	*Pseudomonas straminea*	NRIC0164T	2	-	-	-	-	-	+	-	+	-	-	-	-	-	+	-
[000037]	*Pseudomonas straminea*	NRIC0182	2	-	-	-	-	-	-	-	+	-	-	-	-	-	-	-

*Abbreviations: AJ, Central Research Laboratories, Ajinomoto Co., Kawasaki, Japan; B, Obtained from D. J. Hildebrand, University of California, Berkeley, CA, USA; DSM, Deutsche Sammlung von Microorganismen und Zellkulturen, Braunschweig, Germany; IFO, Institute for Fermentation, Osaka, Japan; NRIC, NODAI Culture Collection, Tokyo University of Agriculture, Tokyo, Japan; T, Type strain.

† Referred from Uchino et al., 2000 (1), Uchino et al., 2001 (2) and unpublished data (3)
